# The Effect of Vitamin D Supplementation on Lipid Profiles: an Umbrella Review of Meta-Analyses

**DOI:** 10.1016/j.advnut.2023.08.012

**Published:** 2023-08-30

**Authors:** Nima Radkhah, Meysam Zarezadeh, Parmida Jamilian, Alireza Ostadrahimi

**Affiliations:** 1 Nutrition Research Center, Tabriz University of Medical Sciences, Tabriz, Iran; 2 Student Research Committee, Department of Clinical Nutrition, Faculty of Nutrition and Food Sciences, Tabriz University of Medical Sciences, Tabriz, Iran; 3 School of Pharmacy and BioEngineering, Keele University, Staffordshire, United Kingdom

**Keywords:** Vitamin D, umbrella meta-analysis, randomized controlled trials, lipid profile, low-density lipoprotein cholesterol, total cholesterol

## Abstract

According to published meta-analyses, vitamin D exerts different beneficial effects in preventing and controlling risk factors associated with noncommunicable chronic diseases; however, the results are still conflicting. The purpose of this umbrella meta-analysis was to investigate the effect of vitamin D supplementation on low-density lipoprotein cholesterol, high-density lipoprotein (HDL) cholesterol, total cholesterol (TC), and triglyceride (TG) as components of the lipid profile. PubMed, Scopus, Web of Science, and Cochrane Database of Systematic Reviews were systematically searched for meta-analyses of randomized controlled trials. The umbrella meta-analysis followed the PRISMA guidelines. The random-effects model was employed to estimate the overall effect size (ES). Overall, 25 meta-analyses were included. In the standardized mean difference analysis, vitamin D significantly decreased TG (ES: −0.15; 95% CI: −0.23, −0.08; *P* ≤ 0.001) and TC levels (ES: −0.17; 95% CI: −0.23, −0.11; *P* ≤ 0.001) and increased HDL levels (ES: 0.08; 95% CI: 0.01, 0.15; *P* = 0.025). In the weighted mean difference analysis, vitamin D significantly decreased only TG levels (ES: −4.63 mg/dL; 95% CI: −7.70, −1.57; *P* = 0.003). The present study supports that vitamin D supplementation could be considered a beneficial adjuvant therapy in managing lipid profile levels, especially in individuals with vitamin D deficiency.

This systematic review was registered in PROSPERO as CRD42022306334.

**Statement of Significance**

Various studies have reported that vitamin D may improve lipid profiles; however, some reported different results. This study, which is the first and the most comprehensive review in this regard, revealed that vitamin D may exert beneficial effects on lipid profiles.

## Introduction

Globally, cardiovascular disease (CVD) is one of the leading causes of morbidity and mortality, with atherosclerosis playing a crucial role in its development [1]. Atherosclerosis begins with vascular endothelium aggression and is precipitated by several risk factors, including dyslipidemias [2]. Dyslipidemia, a significant risk factor for the progression of CVD, is characterized by abnormalities in lipid homeostasis. Therefore, managing dyslipidemia could decrease the likelihood of developing CVD and diabetes [3]. A study evaluating the US population reported that only a 10% increase in the rate of hyperlipidemia treatment would prevent an estimated 8000 deaths annually [4].

In addition to LDL cholesterol, the first lipoprotein of interest, triglyceride (TG), HDL cholesterol, and total cholesterol (TC) play a significant role in CVD, with HDL cholesterol having a potential preventive role and the other lipid profile components having a negative effect [5]. Here, “lipid profile” refers to a collection of lipids including TG, TC, LDL cholesterol, and HDL cholesterol.

Vitamin D is a unique nutrient because it can be obtained either through endogenous synthesis or through diet. Vitamin D, within cells, regulates the transcription of a large and diverse number of genes by binding to its nuclear receptor, the vitamin D receptor (VDR). Through this mechanism, vitamin D not only maintains calcium and phosphate homeostasis and bone mineralization but also regulates cellular growth, differentiation, and immune function, among other vital functions. It may also play a plausible role in cancer, CVD, diabetes, and other diseases [6].

In observational and interventional studies, inadequate vitamin D levels were associated with unfavorable serum lipid profiles, whereas adequate vitamin D levels were associated with favorable lipid profiles [[7], [8], [9], [10]]. A Polish cohort study found an inverse relationship between vitamin D levels and TC, TG, and LDL cholesterol [7]. A significant correlation between an atherogenic lipid profile and vitamin D deficiency was found by analyzing the levels of 25(OH)D and various lipid fractions among 20,000 participants [8]. Recent meta-analyses have also evaluated vitamin D levels, supplementation, and their correlation with the lipid profile [[11], [12], [13]]. A meta-analysis of 8 randomized controlled trials (RCTs) examining the effect of vitamin D supplementation on the lipid profile revealed that vitamin D reduced TG levels and was associated with increasing HDL cholesterol and, interestingly, increasing LDL cholesterol [11]. In a much larger meta-analysis evaluating the pooled effect of vitamin D supplementation on TG, TC, LDL cholesterol, and HDL cholesterol in as many as 39 RCTs, vitamin D significantly decreased TG, TC, and LDL cholesterol levels and increased HDL cholesterol levels [12]. In a larger meta-analysis of 39 RCTs evaluating the effects of vitamin D supplementation on TG, TC, LDL cholesterol, and HDL cholesterol, vitamin D supplementation was found to increase HDL cholesterol levels. Nonetheless, the authors found a statistically significant inverse correlation between vitamin D supplementation and TG, TC, and LDL cholesterol [13].

These findings should be interpreted with caution, considering the number of studies, the heterogeneity of interventions and outcomes, and the methodological quality of the studies. Therefore, given the global prevalence of vitamin D deficiency and the purported beneficial effects of vitamin D on lipid profile, our designed umbrella review of meta-analyses of RCTs aims to evaluate the effects of vitamin D supplements on the lipid profile (TG, TC, LDL cholesterol, and HDL cholesterol) in adults and appraise the existing evidence to inform clinical practice, as well as to highlight additional areas for future research.

## Methods

The protocol of the present study was registered in the PROSPERO database (CRD42022306334). In addition, the present umbrella meta-analysis was reported according to the PRISMA guidelines [14].

### Research objectives

The present umbrella meta-analysis of RCTs was conducted to determine the effect of vitamin D supplementation on the lipid profile. The primary outcomes were TG, TC, LDL cholesterol, and HDL cholesterol levels. There were no additional secondary outcomes.

### Search strategy

We comprehensively searched PubMed, Scopus, Web of Science, and Cochrane Database of Systematic Reviews with the assistance of a librarian experienced in systematic reviews to retrieve relevant meta-analyses. We also searched PROSPERO for related reviews. The references of retrieved articles and existing reviews were manually checked for additional resources. The primary search was conducted from database inception until February 2022, and on September 2022, an additional search was performed to update and locate any potential new studies. Based on the relevant keywords ((“Vitamin D”) AND (TG OR TC OR LDL-c OR HDL-c) AND (“meta-analysis”)), a structured search strategy was determined. The search strategy is detailed in **Supplementary Information**.

### Inclusion criteria

Only meta-analyses of RCTs investigating the effect of vitamin D supplementation on TG, TC, LDL cholesterol, and HDL cholesterol levels in adult male and female subjects aged 18 and older were included in this study. The PICO (Population, Intervention, Comparison, and Outcomes) framework is shown in Table 1. There were no restrictions on vitamin D supplement type, dosage, or duration. We included only English-language articles, with no restrictions on publication date.

**TABLE 1:** PICO for study inclusion

Participants (P)	Intervention (I)	Comparison (C)	Outcomes (O)
**Inclusion criteria**			
Participants ≥18 y	Vitamin D supplementation in the form of vitamin D3 (cholecalciferol) or vitamin D2 (ergocalciferol) at any dose, given orally, daily, weekly, or monthly.	RCTs with the intervention compared to placebo	Triglyceride levels (mg/dL), Total cholesterol levels (mg/dL), LDL-C (mg/dL), and HDL- C (mg/dL)
**Exclusion criteria**			
Participants <18 y	Vitamin D supplements with other vitamin and chemical element supplements; vitamin D supplementation in fortified foods as the amount of vitamin cannot be defined accurately.		

Abbreviations: RCT, randomized controlled trial.

### Exclusion criteria

We excluded any other type of study than meta-analyses of RCTs. We also excluded those studies that lacked relevant data. Studies that investigated the effect of vitamin D on children were excluded. Moreover, meta-analyses evaluating the effect of vitamin D in combination with cosupplements were excluded. However, meta-analyses that did not intend to evaluate a combination of vitamin D with cosupplements but have included one or 2 such studies were included. Studies with vitamin D supplementation in fortified foods, as the amount of vitamin cannot be defined accurately, were excluded.

### Study screening and inclusion

Two independent reviewers (NR, MZ) screened all retrieved articles through titles and abstracts. If there was any uncertainty regarding whether a study should be excluded, the study was advanced to the full-text screen to reduce the likelihood of being erroneously excluded. Two reviewers obtained the full text of potentially relevant papers for an independent analysis. A third party (AO) reconciled any disagreements. Articles excluded in the full-text screening phase were reported with respective reasons. We completed a PRISMA flowchart to summarize this process, and a PRISMA checklist is also appended.

### Data extraction

Included studies underwent a standardized data extraction process employing a preformatted spreadsheet by one of the authors (NR). A second reviewer (MZ) verified the extracted data to reduce reviewer errors and bias. In the case of missing data from reports, we attempted to contact the study authors to get the necessary information. Where an included study had more than 2 comparisons, only the ones that met the eligibility criteria were considered. The following details were extracted: first author, year of publication, study design, type of study, sample size, length of follow-up, type and dosage of vitamin D supplements, age, sex, and effect size (ES) and their corresponding confidence intervals (CIs) for TG, TC, LDL cholesterol, and HDL cholesterol.

### Quality assessment

Two review authors (NR, MZ) independently assessed the quality of meta-analyses according to the AMSTAR 2 tool [15]. This tool contains 16 items, of which 7 are critical domains that can critically affect the validity of a review and its conclusions (Items 2, 4, 7, 9, 11, 13, and 15).

### Data synthesis and statistical analysis

The reported ESs and CIs were used to estimate the overall ES. We pooled estimates of treatment effects where possible, using standard statistical techniques. The random-effects model was applied to conduct statistical analysis using the restricted maximum likelihood method. Due to the natural differences between standardized mean difference (SMD) and weighted mean difference (WMD), the analysis was performed for each separately. We used the chi-squared test with a significance level of *P* < 0.10 in conjunction with the I^2^ measure to evaluate statistical heterogeneity [16]. The I^2^ test assesses the percentage of total variation across studies due to heterogeneity rather than chance [16]. Values greater than 75% were generally considered to indicate the presence of high heterogeneity, and values of 40% or less indicated low heterogeneity [16]. We explored further if there was evidence of high heterogeneity. We assessed potential sources of heterogeneity by performing subgroup analyses. We considered the following subgroups: age groups, sex, health status, vitamin D dose and duration, number of included ESs, quality of studies, and using cosupplements (studies that included one or more articles using cosupplements with vitamin D). Sensitivity analysis was conducted to explore each study’s removal influence on overall results and assess the robustness of the results. All statistical analyses were carried out using STATA version 17.0 (Stata Corporation). *P* value < 0.05 was considered a significant level.

### Assessment of bias

Reporting bias arises when the nature and direction of results influence the dissemination of research findings. Publication bias is one of several possible causes of small-study effects, which tends to estimate the effect of an intervention to be more beneficial in smaller studies.

We used funnel plots to assess small-study effects visually [17], along with Egger’s [18] and Begg’s tests [19]. The publication bias assessment was done when at least 8 studies were included for each primary outcome. In case of publication bias, we performed the trim-and-fill analysis to present a new ES by stimulating a model without publication bias.

## Results

### Systematic review

**FIGURE 1:**
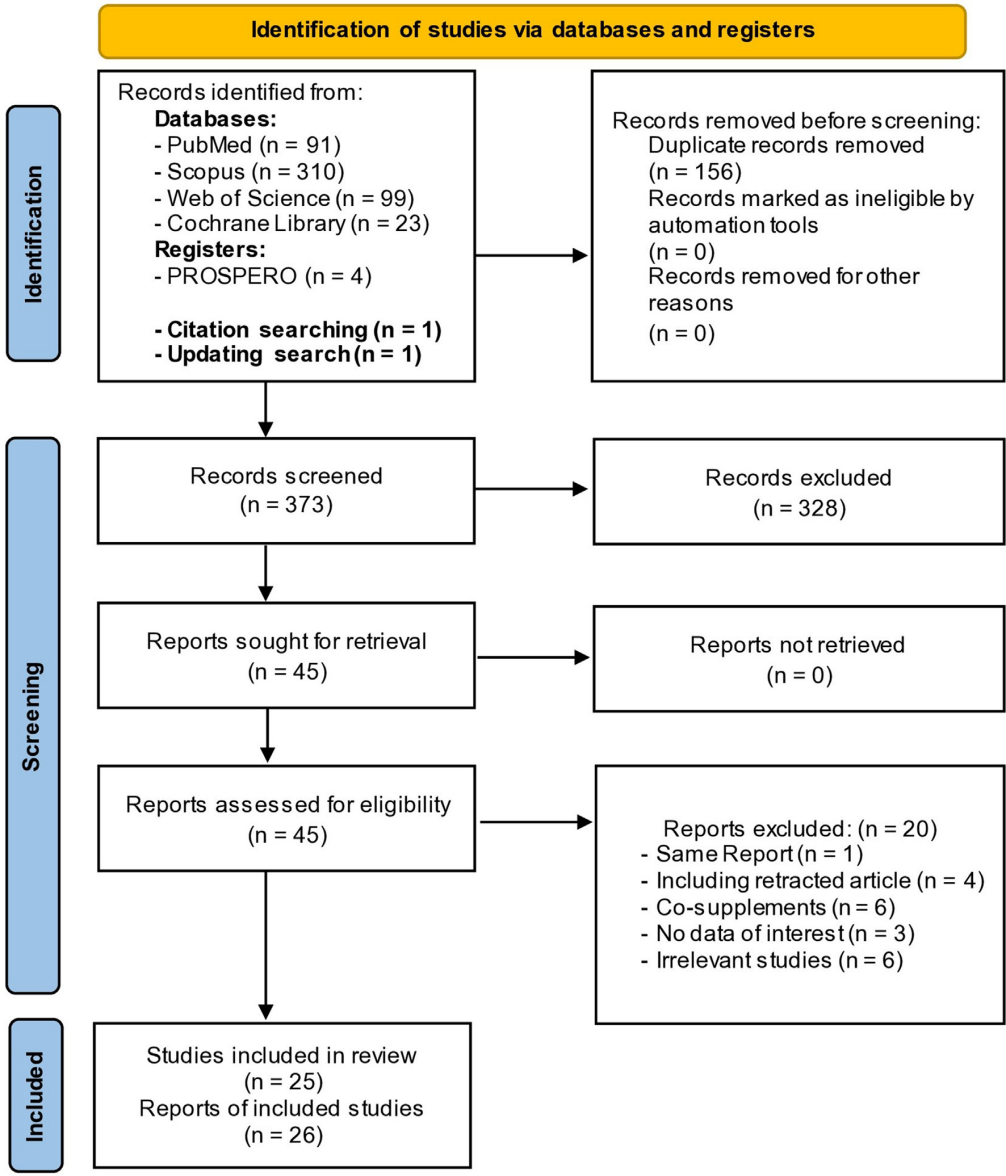
Flow diagram of study selection.

The flow diagram of the literature search process is summarized in Figure 1. Overall, 25 meta-analyses with 26 ESs published between 2012 and 2022 were included in this meta-analysis [11,12,[20], [21], [22], [23], [24], [25], [26], [27], [28], [29], [30], [31], [32], [33], [34], [35], [36], [37], [38], [39], [40], [41], [42]]. Three hundred twenty-eight studies were excluded during the title and abstract screening. In the full-text screen, we excluded 4 studies [[43], [44], [45], [46]] because they had a retracted RCT [47]. In addition, we found two studies with identical reports [28,48]; we only included the latest one. Six studies were excluded because of investigating a combination of vitamin D with cosupplements [13,[49], [50], [51], [52]]. Three studies had no data of interest [41,53,54], and 6 were irrelevant to our aim [24,[55], [56], [57], [58], [59]].

The characteristics of the included studies are presented in Table 2. The mean age of the studies’ participants was between 25 and 68 y. The duration of interventions varied from 3 to 260 wk. The dose of the intervention varied from 20 to 50,000 IU/d. Six studies evaluated the effect of vitamin D on lipid profile in individuals with polycystic ovary syndrome (PCOS) [25,27,29,31,32,40], 4 studies in patients with nonalcoholic fatty liver disease [26,37,38,41], 3 studies in individuals with CVD [21,22,35], 2 in individuals with diabetes [28,42], 1 in individuals with metabolic syndrome [20], 1 in individuals with chronic kidney disease [34], 1 in postmenopausal women [30], 1 in individuals with obesity [11], and other studies with no specific statistical population [12,23,24,33,36,39]. Moreover, the number of included studies in the investigated meta-analyses varied from 2 to 38 studies.

**TABLE 2:** Study characteristics of included studies

First author, date, and location	Included studies (*n*)	Participants (*n*) and health condition	Age (y) and sex	Intervention (Range and mean of dose and duration)	Results	Quality
Milajerdi A (2019) [34] Iran	6	333 CKD	48.6 M/F	20–50,000 IU/d (14,590.95 IU/d) 3–16 wk (10.5 wk)	TG ^S ↓^ TC ^S ↓^ LDL-C ^NS^ HDL-C ^NS^	Yes (Cochrane) 6/6 High
Jin B (2020) [29] China	9	437 PCOS	26.28 F	2500–12,000 IU/d (5507.89 IU/d) 8-24 wk (11.11 wk)	TG ^S ↓^ TC ^S ↓^ LDL-C ^S ↓^ HDL-C ^NS^	Yes (Cochrane) 8/9 High
Wang H (2012) [39] China	9	1188 Healthy, Obesity, and Diabetes	51.81 M/F	20–8571 IU/d (2753.13 IU/d) 6–144 wk (57.25 wk)	TG ^NS^ TC ^NS^ LDL-C ^S ↓^ HDL-C ^NS^	Yes (Jadad) 7/9 High
Bahrami LS (2020) [21] Iran	3	209 Coronary Artery Disease	61.46 M/F	20–7143 IU/d (3578.10 IU/d) 8–24 wk (18.66 wk)	TG ^NS^ TC ^NS^ LDL-C ^NS^ HDL-C ^NS^	Yes (Cochrane) 2/3 Moderate
Miao YC (2020) [32] China	5	217 PCOS	26.69 F	3571–12,000 IU/d (6056.97 IU/d) 8–24 wk (12.8 wk)	TG ^NS^ TC ^S ↓^ LDL-C ^S ↓^ HDL-C ^NS^	Yes (Cochrane) 3/5 High
Rezaei S (2021) [37] Iran	8	685 NAFLD	NA M/F	10–7143 IU/d (4082.43 IU/d) 10–48 wk (16.2 wk)	TG ^NS^ TC ^NS^ LDL-C ^NS^ HDL-C ^S ↑^	Yes (Cochrane) 5/8 High
Gao H (2021) [25] China	10	543 PCOS	26.72 F	2500–12,000 IU/d (4848.51 IU/d) 8–24 wk (12.8 wk)	TG ^S ↓^ TC ^S ↓^ LDL-C ^S ↓^ HDL-C ^NS^	Yes (Cochrane) 5/10 High
Jafari T (2016) [28] Iran	14	1044 T2DM	57.42 M/F	1000–7143 IU/d (3229.67 IU/d) 8–24 wk (17.84 wk)	TG ^NS^ TC ^S ↓^ LDL-C ^S ↓^ HDL-C ^S ↓^	Yes (Jadad) 8/14 High
Zou Y (2021) [42] China	24	674 Diabetes and Prediabetes	NA M/F	NA NA	TG ^NS^ TC ^NS^ LDL-C ^S ↓^ HDL-C ^S ↑^	Yes (Cochrane) 18/24 High
AlAnouti F (2020) [20] United Arab Emirates	3	105 MS	52.95 M/F	2000–2857 IU/d (2428.57 IU/d) 8–12 wk (10 wk)	TG ^S ↑^ TC ^NS^ LDL-C ^NS^ HDL-C ^NS^	Yes (Cochrane) 1/3 High
AlAnouti F (2020) [20] United Arab Emirates	2	127 MS	52.08 M/F	5714–7143 IU/d (6428.57 IU/d) 8–16 wk (12 wk)	TG ^S ↑^ TC ^NS^ LDL-C ^NS^ HDL-C ^NS^	Yes (Cochrane) 2/2 High
Guo XF (2020) [26] China	4	347 NAFLD	44.6 M/F	10–7143 IU/d (3065.95 IU/d) 10–48 wk (17.6 wk)	TG ^NS^ TC ^NS^ LDL-C ^NS^ HDL-C ^NS^	Yes (Cochrane) 0/4 High
Liu W (2021) [30] China	7	1109 Postmenopausal	55.98 F	300–4000 IU/d (1181.81 IU/d) 12–144 wk (46.36 wk)	TG ^S ↓^ TC ^NS^ LDL-C ^NS^ HDL-C ^S ↓^	Yes (Cochrane) 6/7 High
Ostadmohammadi V (2019) [35] Iran	5	343 Cardiovascular Disease	63.87 M/F	20–7142.86 IU/d (2941.14 IU/d) 8–24 wk (16 wk)	TG ^NS^ TC ^NS^ LDL-C ^NS^ HDL-C ^S ↑^	Yes (Cochrane) NR
Bjelakovic M (2021) [22] Serbia	5	460 Chronic Liver Diseases	44.3 M/F	10–7143 IU/d (4172.44 IU/d) 10–24 wk (14 wk)	TG ^NS^ TC ^NS^ LDL-C ^NS^ HDL-C ^NS^	Yes (Cochrane) 3/5 High
Elamin MB (2011) [24] United States	12	2098 General	NA M/F	NA	TG ^NS^ TC ^NS^ LDL-C ^NS^ HDL-C ^NS^	NA
Wang L (2020) [40] China	7	401 PCOS	27.58 F	2500–12,000 IU/d (4977.55 IU/d) 8–24 wk (12 wk)	TG ^NS^ TC ^S ↓^ LDL-C ^NS^ HDL-C ^NS^	Yes (Cochrane) 6/7 High
Dibaba DT (2019) [23] United States	34	3242 General	55 M/F	20–7143 IU/d (2616.76 IU/d) 8–144 wk (32.41 wk)	TG ^NS^ TC ^S ↓^ LDL-C ^S ↓^ HDL-C ^NS^	Yes (Jadad) Most High
Tabrizi R (2017) [38] Iran	4	279 NAFLD	44.08 M/F	1000–7143 IU/d (2785.72 IU/d) 10–12 wk (11.5 wk)	TG ^NS^ TC ^NS^ LDL-C ^NS^ HDL-C ^NS^	Yes (Cochrane) 1/4 High
Luo J (2021) [31] China	12	677 PCOS	25.99 F	400–12,000 IU/d (4485.68 IU/d) 8–24 wk (12 wk)	TG ^S ↓^ TC ^S ↓^ LDL-C ^S ↓^ HDL-C ^NS^	Yes (Cochrane) 7/12 High
Qorbani M (2022) [36] Iran	10	983 General	67.65 M/F	400–14712 IU/d (4276.78 IU/d) 8–48 wk (25.77 wk)	TG ^S ↓^ TC ^S ↓^ LDL-C ^NS^ HDL-C ^NS^	Yes (CONSORT) 6/10 High
He C (2015) [27] United States	3	130 PCOS	26.76 F	2500–12,000 IU/d (7214.33 IU/d) 8–12 wk (9.33 wk)	TG ^NS^ LDL-C ^NS^ HDL-C ^NS^	Yes (PRISMA) NR
Mirhosseini N (2018) [12] Canada	39	3693 General	49.49 M/F	300–12,000 IU/d (2978.74 IU/d) 12–240 wk (31.94 wk)	TG ^S ↓^ TC ^S ↓^ LDL-C ^S ↓^ HDL-C ^S ↑^	Yes (Cochrane) Most High
Manousopoulou A (2015) [11] United Kingdom	5	755 Obesity	45 M/F	1000–8571.5 IU/d (4294.97 IU/d) 6–48 wk (32.4 wk)	TG ^S ↓^ LDL-C ^S ↑^ HDL-C ^NS^	Yes (Jadad) 1/5 High
Miao J (2021) [33] United States	20	3098 General	44.89 M/F	400–12,000 IU/d (4754.67 IU/d) 8–260 wk (33.3 wk)	LDL-C ^NS^	NR
Wei Y (2020) [41] China	4	269 NAFLD	47.05 M/F	10–7143 IU/d (2450.57 IU/d) 12–48 wk (21.6 wk)	LDL-C ^NS^ HDL-C ^NS^	Yes (Cochrane) 3/4 High

Abbreviations: CKD, chronic kidney disease; F, female; HDL-C, high-density lipoprotein cholesterol; LDL-C, low-density lipoprotein cholesterol; M, male; MS, metabolic syndrome; NA, not available; NAFLD, nonalcoholic fatty liver disease; NR, not reported; NS, nonsignificant; PCOS, polycystic ovary syndrome; S, significant; T2DM, type 2 diabetes mellitus; TC, total cholesterol; TG, triglyceride.

### RoB assessment

The results of the quality assessment of meta-analyses according to the AMSTAR2 questionnaire are summarized in Table 3. Almost all of the included meta-analyses in the umbrella review were evaluated as low and critically low-quality studies. Only one study was assessed as moderate quality [39] and 2 as high quality [12,22]. Item 7, as a major domain in the AMSTAR2, was the most frequent item that was not adhered to in most of the articles and was the most important reason for lowering the evaluated quality scores. In this item, authors are required to provide a list of excluded studies and justify the exclusions.

**TABLE 3:** Results of assessment of the methodological quality of the meta-analysis

First author	Q1	Q2	Q3	Q4	Q5	Q6	Q7	Q8	Q9	Q10	Q11	Q12	Q13	Q14	Q15	Q16	Overall
Milajerdi A [34]	Y	P Y	Y	P Y	Y	Y	N	Y	Y	N	Y	Y	Y	Y	Y	Y	Low
Wang L [40]	Y	P Y	Y	P Y	N	Y	N	Y	Y	N	Y	Y	Y	N	Y	Y	Low
Dibaba DT [23]	Y	P Y	Y	P Y	N	N	N	Y	P Y	N	Y	N	Y	Y	Y	Y	Low
Jin B [29]	Y	P Y	Y	P Y	Y	Y	N	Y	Y	N	Y	Y	Y	Y	Y	Y	Low
Wang H [39]	Y	P Y	Y	P Y	N	Y	Y	Y	P Y	N	Y	Y	Y	Y	Y	Y	Moderate
Sadat Bahrami L [21]	Y	P Y	Y	P Y	N	Y	N	Y	Y	N	N	N	Y	Y	Y	Y	Critically Low
Miao CY [32]	Y	P Y	Y	P Y	N	Y	N	Y	Y	N	Y	Y	Y	Y	Y	Y	Low
Mirhosseini N [12]	Y	Y	Y	P Y	Y	Y	P Y	Y	Y	N	Y	Y	Y	Y	Y	Y	High
Rezaei S [37]	Y	P Y	Y	P Y	Y	Y	N	Y	Y	N	Y	Y	Y	Y	Y	Y	Low
Gao H [25]	Y	P Y	Y	P Y	Y	Y	N	N	Y	N	Y	Y	Y	Y	Y	Y	Low
Jafari T [28]	Y	Y	Y	P Y	Y	Y	N	P Y	P Y	N	Y	N	N	Y	Y	Y	Critically Low
Zou Y [42]	Y	Y	Y	P Y	N	Y	N	Y	Y	N	Y	Y	Y	Y	Y	Y	Low
AlAnouti F [20]	Y	Y	Y	Y	Y	Y	N	Y	Y	N	Y	Y	Y	Y	N	Y	Critically Low
Tabrizi R [38]	Y	P Y	Y	P Y	Y	Y	N	P Y	Y	N	N	Y	Y	Y	Y	Y	Critically Low
Guo XF[26]	Y	P Y	Y	P Y	N	Y	N	Y	Y	N	Y	Y	Y	Y	Y	Y	Low
Luo J [31]	Y	P Y	Y	P Y	Y	Y	N	Y	Y	N	Y	Y	Y	Y	Y	Y	Low
Manousopoulou A [11]	Y	P Y	Y	P Y	N	N	Y	Y	P Y	N	N	N	N	N	Y	Y	Critically Low
Liu W [30]	Y	P Y	Y	P Y	N	Y	N	Y	Y	N	Y	Y	Y	Y	Y	Y	Low
Ostadmohammadi V [35]	Y	P Y	Y	P Y	Y	N	N	Y	Y	N	Y	Y	Y	Y	Y	Y	Low
Bjelakovic M [22]	Y	Y	Y	Y	Y	Y	Y	Y	Y	Y	Y	Y	Y	Y	Y	Y	High
Elamin MB [24]	Y	P Y	Y	Y	N	Y	N	P Y	P Y	Y	Y	Y	Y	Y	N	Y	Critically Low
Miao J [33]	Y	N	Y	P Y	N	N	N	Y	N	N	Y	N	N	Y	N	Y	Critically Low
He C [27]	Y	P Y	N	P Y	Y	N	N	P Y	N	N	Y	Y	Y	Y	Y	Y	Critically Low
Wei Y [41]	Y	P Y	Y	P Y	Y	Y	N	Y	Y	N	Y	Y	Y	Y	Y	Y	Low
Qorbani M [36]	Y	P Y	Y	P Y	Y	Y	N	Y	Y	N	Y	Y	Y	Y	Y	Y	Low

Q1- Did the research questions and inclusion criteria for the review include the components of PICO? Q2- Did the report of the review contain an explicit statement that the review methods were established prior to the conduct of the review, and did the report justify any significant deviations from the protocol? Q3- Did the review authors explain their selection of the study designs for inclusion in the review? Q4- Did the review authors use a comprehensive literature search strategy? Q5- Did the review authors perform study selection in duplicate? Q6- Did the review authors perform data extraction in duplicate? Q7- Did the review authors provide a list of excluded studies and justify the exclusions? Q8- Did the review authors describe the included studies in adequate detail? Q9- Did the review authors use a satisfactory technique for assessing risk of bias (RoB) in individual studies that were included in the review? Q10- Did the review authors report on the sources of funding for the studies included in the review? Q11- If meta-analysis was performed, did the review authors use appropriate methods for the statistical combination of results? Q12- If meta-analysis was performed, did the review authors assess the potential impact of RoB in individual studies on the results of the meta-analysis or other evidence synthesis? Q13- Did the review authors account for RoB in individual studies when interpreting/discussing the review results? Q14- Did the review authors provide a satisfactory explanation for and discussion of any heterogeneity observed in the review results? Q15- If they performed quantitative synthesis, did the review authors conduct an adequate investigation of publication bias (small-study bias) and discuss its likely impact on the review results? Q16- Did the review authors report any potential sources of conflict of interest, including any funding they received for conducting the review? Abbreviations: Y, Yes; PY, Partially Yes; N, No.

### Effect of vitamin D supplementation on TG levels

#### Effect of vitamin D supplementation on TG levels in studies reporting SMD

**FIGURE 2:**
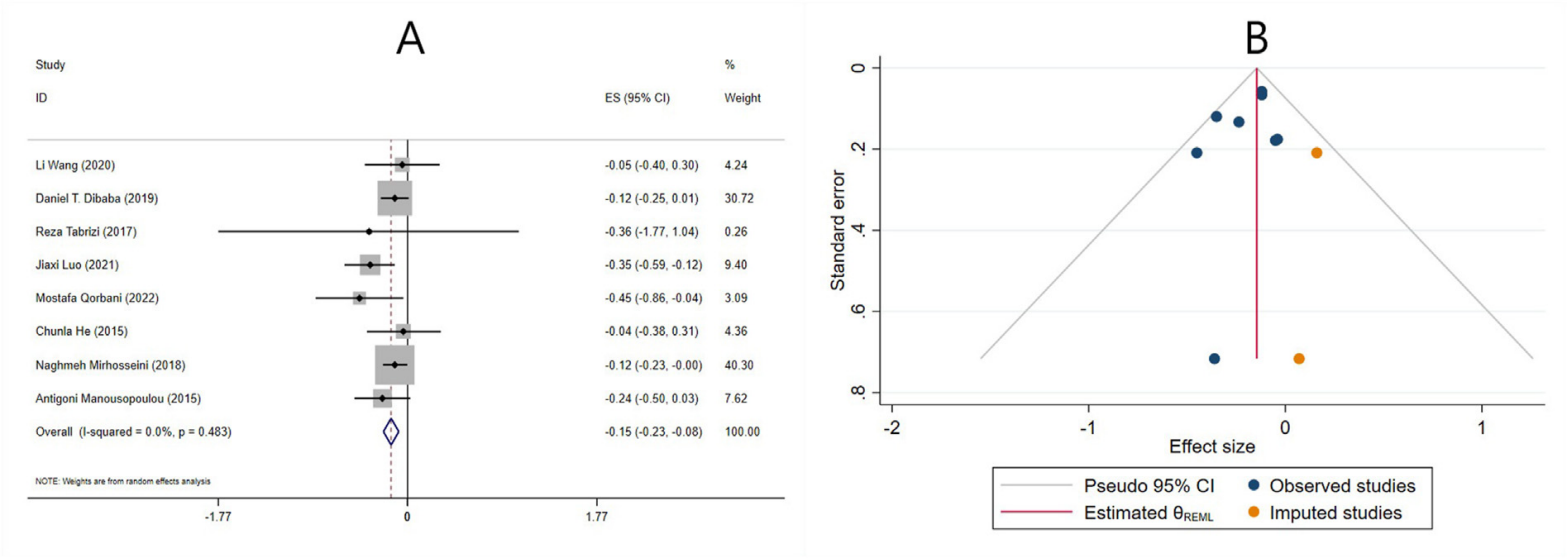
The effects of vitamin D supplementation on triglyceride levels. Forest plot (A) detailing standardized mean difference (SMD) and 95% confidence intervals (CIs); Trim-and-fill analysis (B). ES, effect size; REML, restricted maximum likelihood method.

The effect of vitamin D supplementation on TG levels was reported in 8 meta-analyses reporting SMD. The combined ESs from these studies demonstrated a significant reduction in TG levels (ES: −0.15; 95% CI: −0.23, −0.08; *P* ≤ 0.001) (Figure 2A). There was no significant heterogeneity between studies (I^2^ = 0.0%, *P* = 0.483) (Figure 2A). Subgroup analysis revealed that vitamin D supplementation significantly reduced TG levels in studies that included both sexes, had participants ≤50 y old, women without PCOS, individuals without renal and liver disease, low-quality studies, and in meta-analyses that did not include any studies with cosupplements. Moreover, the effect of vitamin D in decreasing TG levels was more pronounced in meta-analyses with <10 included ESs, >4000 IU/d supplementation dose, and ≤14 wk of intervention (Table 4). Furthermore, subgroup analysis was performed to investigate the impact of renal and liver disease on overall ES. After excluding studies that evaluated liver and renal disease, the result remained unchanged, and vitamin D significantly reduced TG levels (Table 4).

**TABLE 4:** Subgroup analyses for the effects of vitamin D supplementation on lipid profile

	Effect size number	ES (95% CI)^1^	*P*-within^2^	I^2^ (%)^3^	*P*-heterogeneity^4^
**Effect of vitamin D supplementation on TG levels (SMD)**					
Overall	8	-0.15 (-0.23, -0.08)	≤ 0.001	0.0	0.483
**Renal and liver disease**					
Yes	1	-0.36 (-1.76, 1.04)	0.421	—	—
No	7	-0.15 (-0.22, -0.08)	0.014	0.0	0.378
**Number of included studies**					
<10	5	-0.19 (-0.35, -0.02)	0.024	0.0	0.548
≥10	3	-0.16 (-0.27, -0.05)	0.004	38.5	0.197
**Sex**					
F	3	-0.18 (-0.40, 0.04)	0.104	35.4	0.213
M/F	5	-0.14 (-0.22, -0.06)	≤ 0.001	0.0	0.556
**Age (y)**					
≤50	6	-0.16 (-0.25, -0.07)	≤ 0.001	0.0	0.516
>50	2	-0.23 (-0.53, 0.08)	0.143	55.8	0.133
**Study population**					
PCOS	3	-0.18 (-0.40, 0.04)	0.104	35.4	0.213
Other	5	-0.14 (-0.22, -0.06)	≤ 0.001	0.0	0.556
**Dose (IU/d)**					
>4000	5	-0.24 (-0.38, -0.10)	≤ 0.001	6.4	0.370
≤4000	3	-0.12 (-0.21, -0.04)	0.005	0.0	0.946
**Duration (wk)**					
≤14	4	-0.20 (-0.38, -0.03)	0.024	4.5	0.370
>14	4	-0.14 (-0.22, -0.06)	≤ 0.001	0.0	0.404
**Presence of cosupplementation**					
No	6	-0.15 (-0.25, -0.04)	0.005	0.0	0.629
Yes	2	-0.21 (-0.43, 0.01)	0.061	66.5	0.084
**Quality**					
Critically low	3	-0.17 (-0.37, 0.04)	0.108	0.0	0.650
Low	4	-0.21 (-0.37, -0.05)	0.010	40.3	0.170
High	1	-0.12 (-0.23, -0.01)	0.038	—	—
**Effect of vitamin D supplementation on TG levels (WMD)**					
Overall	16	-4.63 (-7.70, -1.57)	0.003	57.0	0.003
**Renal and liver disease**					
Yes	4	-6.74 (-21.50, 8.01)	0.677	68.75	0.042
No	12	-4.79 (-7.32, -2.25)	0.006	39.80	0.005
**Number of included studies**					
<10	10	-3.17 (-9.09, 2.74)	0.293	70.6	≤ 0.001
≥10	6	-4.53 (-6.46, -2.60)	≤ 0.001	0.0	0.555
**Sex**					
F	4	-7.07 (-10.98, -3.16)	≤ 0.001	67.3	0.027
M/F	12	-2.44 (-7.20, 2.32)	0.316	53.0	0.016
**Age(y)**					
≤50	6	-8.92 (-13.49, -4.35)	≤ 0.001	44.3	0.110
>50	7	-1.19 (-5.81, 3.43)	0.615	56.1	0.034
NR	3	-3.91 (-9.05, 1.24)	0.137	0.0	0.533
**Dose (IU/d)**					
>4000	7	-5.50 (-12.03, 1.02)	0.098	67.4	0.005
≤4000	7	-3.17 (-6.71, 0.37)	0.079	38.2	0.137
NR	2	-4.71 (-10.30, 0.87)	0.098	0.0	0.394
**Duration (wk)**					
≤14	7	-3.19 (-11.31, 4.93)	0.442	76.4	≤ 0.001
>14	7	-3.61 (-5.57, -1.65)	≤ 0.001	0.0	0.767
NR	2	-4.71 (-10.30, 0.87)	0.098	0.0	0.394
**Presence of cosupplementation**					
No	11	-4.54 (-8.62, -0.47)	0.029	68.9	≤ 0.001
Yes	3	-3.92 (-9.92, 2.08)	0.200	0.0	0.381
NR	2	-4.71(-10.30, 0.87)	0.098	0.0	0.394
**Quality**					
Critically low	5	3.46 (-5.82, 12.74)	0.465	66.5	0.018
Low	9	-7.24 (-10.45, -4.03)	≤ 0.001	48.5	0.050
Moderate	1	-1.92 (-7.72, 3.88)	0.516	—	—
High	1	11.27 (-10.99, 33.53)	0.321	—	—
**Effect of vitamin D supplementation on TC levels (SMD)**					
Overall	7	-0.17 (-0.23, -0.11)	≤ 0.001	0.0	0.719
**Renal and liver disease**					
Yes	2	-0.16 (-0.40, 0.07)	0.112	0.0	0.479
No	5	-0.17 (-0.23, -0.11)	0.041	0.0	0.528
**Number of included studies**					
<10	3	-0.23 (-0.42, -0.04)	0.016	0.0	0.528
≥10	4	-0.16 (-0.22, -0.10)	≤ 0.001	0.0	0.585
**Sex**					
F	2	-0.35 (-0.55, -0.14)	≤ 0.001	0.0	0.962
M/F	5	-0.15 (-0.22, -0.09)	≤ 0.001	0.0	0.957
**Age(y)**					
≤50	4	-0.27 (-0.43, -0.11)	≤ 0.001	0.0	0.627
>50	3	-0.15 (-0.22, -0.09)	≤ 0.001	0.0	0.931
**Dose (IU/d)**					
>4000	2	-0.35 (-0.55, -0.14)	≤ 0.001	0.0	0.962
≤4000	5	-0.15 (-0.22, -0.09)	≤ 0.001	0.0	0.957
**Duration (wk)**					
≤14	4	-0.27 (-0.43, -0.11)	≤ 0.001	0.0	0.627
>14	3	-0.15 (-0.22, -0.09)	≤ 0.001	0.0	0.931
**Presence of cosupplementation**					
No	5	-0.17 (-0.24, -0.09)	≤ 0.001	0.0	0.745
Yes	2	-0.21 (-0.38, -0.03)	0.022	41.8	0.190
**Quality**					
Critically low	1	-0.46 (-1.31, 0.39)	0.289	—	—
Low	5	-0.18 (-0.25, -0.11)	≤ 0.001	0.0	0.548
High	1	-0.15 (-0.25, -0.06)	0.005	—	—
**Effect of vitamin D supplementation on TC levels (WMD)**					
Overall	15	-2.87 (-5.93, 0.19)	0.066	84.2	≤ 0.001
**Renal and liver disease**					
Yes	3	-0.19 (-8.76, 8.37)	0.742	78.82	0.006
No	12	-3.42 (-6.76, -0.07)	0.048	85.28	≤ 0.001
**Number of included studies**					
<10	9	-3.02 (-7.85, 1.81)	0.220	80.3	≤ 0.001
≥10	6	-2.57 (-6.76, 1.61)	0.228	88.5	≤ 0.001
**Sex**					
F	11	-7.89 (-14.06, -1.72)	0.012	89.7	≤ 0.001
M/F	4	-0.70 (-3.00, 1.60)	0.552	52.7	0.020
**Age(y)**					
≤50	5	-7.83 (-12.35, -3.30)	≤ 0.001	79.3	≤ 0.001
>50	7	-0.24 (-2.61, 2.13)	0.845	39.0	0.132
NR	3	0.08 (-2.25, 2.41)	0.945	0.0	0.428
**Dose (IU/d)**					
>4000	5	-9.23 (-12.62, -5.85)	≤ 0.001	56.4	0.057
≤4000	8	0.18 (-2.07, 2.43)	0.874	38.5	0.123
NR	2	-0.25 (-2.64, 2.15)	0.840	0.0	0.524
**Duration (wk)**					
≤14	6	-9.50 (-12.71, -6.29)	≤ 0.001	47.9	0.088
>14	7	0.31 (-1.72, 2.34)	0.766	29.9	0.200
NR	2	-0.25 (-2.64, 2.15)	0.840	0.0	0.524
**Presence of cosupplementation**					
No	10	-4.19 (-8.60, 0.22)	0.062	87.5	≤ 0.001
Yes	3	-0.11 (-5.06, 4.84)	0.965	60.2	0.081
NR	2	-0.25 (-2.64, 2.15)	0.840	0.0	0.524
**Quality**					
Critically low	5	-1.28 (-4.11, 1.55)	0.376	42.3	0.139
Low	8	-4.99 (-9.49, -0.50)	0.030	84.2	≤ 0.001
Moderate	1	1.52 (-1.42, 4.46)	0.311	—	—
High	1	3.51 (-2.83, 9.85)	0.278	—	—
**Effect of vitamin D supplementation on LDL levels (SMD)**					
Overall	9	-0.10 (-0.20, 0.00)	0.053	57.9	0.015
**Renal and liver disease**					
Yes	2	-0.25 (-0.99, 0.48)	0.534	68.97	0.073
No	7	-0.10 (-0.22, 0.01)	0.059	69.37	0.019
**Number of included studies**					
<10	6	-0.05 (-0.24, 0.14)	0.609	63.5	0.018
≥10	3	-0.15 (-0.24, -0.05)	0.002	40.3	0.187
**Sex**					
F	3	-0.22 (-0.35, -0.09)	≤ 0.001	0.0	0.550
M/F	6	-0.05 (-0.18, 0.07)	0.404	65.2	0.013
**Age**					
≤50	6	-0.08 (-0.30, 0.14)	0.466	72.9	0.002
>50	3	-0.12 (-0.19, -0.05)	≤ 0.001	0.0	0.812
**Study population**					
PCOS	3	-0.22 (-0.35, -0.09)	≤ 0.001	0.0	0.550
NAFLD	4	-0.26 (-0.99, 0.48)	0.495	69.0	0.073
Other	2	-0.05 (-0.19, 0.09)	0.474	72.4	0.012
**Dose (IU/d)**					
>4000	4	-0.03 (-0.26, 0.21)	0.817	71.2	0.015
≤4000	5	-0.13 (-0.24, -0.03)	0.009	46.1	0.115
**Duration (wk)**					
≤14	4	-0.23 (-0.36, -0.10)	≤ 0.001	0.0	0.429
>14	5	-0.04 (-0.16, 0.08)	0.507	66.2	0.019
**Presence of cosupplementation**					
No	7	-0.06 (-0.20, 0.08)	0.384	58.9	0.024
Yes	2	-0.18 (-0.35, -0.00)	0.048	69.0	0.073
**Quality**					
Critically low	3	-0.06 (-0.55, 0.44)	0.824	76.7	0.014
Low	5	-0.15 (-0.23, -0.06)	≤ 0.001	18.6	0.296
High	1	-0.10 (-0.20, -0.00)	0.047	—	—
**Effect of vitamin D supplementation on LDL Levels (WMD)**					
Overall	17	-1.69 (-3.66, 0.28)	0.092	75.5	≤ 0.001
**Renal and liver disease**					
Yes	4	0.79 (-2.11, 3.71)	0.253	8.92	0.152
No	13	-2.13 (-4.18, -0.07)	0.036	75.53	≤ 0.001
**Number of included studies**					
<10	12	-1.04 (-3.67, 1.60)	0.441	71.0	≤ 0.001
≥10	5	-2.81 (-5.83, 0.20)	0.067	81.5	≤ 0.001
**Sex**					
F	4	-3.27 (-6.31, -0.24)	0.034	73.9	0.009
M/F	13	-1.01 (-3.60, 1.58)	0.444	76.9	≤ 0.001
**Age**					
≤50	7	-3.33 (-5.21, -1.45)	≤ 0.001	32.1	0.183
>50	7	0.62 (-1.78, 3.02)	0.613	55.9	0.034
NR	3	-2.44 (-9.37, 4.48)	0.489	88.7	≤ 0.001
**Dose (IU/d)**					
>4000	6	-4.07 (-5.63, -2.51)	≤ 0.001	0.0	0.419
≤4000	9	0.81 (-1.09, 2.70)	0.402	48.7	0.049
NR	2	-6.39 (-8.91, -3.88)	≤ 0.001	13.0	0.284
**Duration (wk)**					
≤14	6	-4.78 (-6.54, -3.02)	≤ 0.001	0.0	0.609
>14	9	0.53 (-1.16, 2.22)	0.535	48.0	0.052
NR	2	-6.39 (-8.91, -3.88)	≤ 0.001	13.0	0.284
**Presence of cosupplementation**					
No	12	-1.19 (-3.50, 1.12)	0.313	70.8	≤ 0.001
Yes	3	-0.77 (-4.11, 2.57)	0.652	46.1	0.156
NR	2	-6.39 (-8.91, -3.88)	≤ 0.001	13.0	0.284
**Quality**					
Critically low	6	-2.16 (-3.87, -0.45)	0.013	0.0	0.672
Low	9	-2.30 (-5.06, 0.47)	0.104	81.6	≤ 0.001
Moderate	1	3.23 (0.55, 5.91)	0.018	—	—
High	1	-0.97 (-8.70, 6.76)	0.806	—	—
**Effect of vitamin D supplementation on HDL levels (SMD)**					
Overall	9	0.08 (0.01, 0.15)	0.025	9.0	0.360
**Renal and liver disease**					
Yes	2	0.23 (-0.01, 0.48)	0.062	0.00	0.782
No	7	0.06 (0.00, 0.13)	0.050	0.01	0.316
**Number of included studies**					
5	6	0.14 (0.03, 0.24)	0.011	0.0	0.776
10	3	0.01 (-0.13, 0.15)	0.908	56.5	0.100
**Sex**					
F	3	0.04 (-0.11, 0.19)	0.590	0.0	0.677
M/F	6	0.09 (-0.01, 0.19)	0.088	35.2	0.173
**Age (y)**					
≤40	7	0.09 (0.03, 0.15)	0.005	0.0	0.853
>40	2	0.05 (-0.47, 0.57)	0.849	79.0	0.029
**Study population**					
PCOS	3	0.04 (-0.11, 0.19)	0.590	0.0	0.677
NAFLD	2	0.24 (-0.01, 0.48)	0.060	0.0	0.782
Other	4	0.06 (-0.06, 0.18)	0.319	50.8	0.107
**Dose (IU/d)**					
>4000	3	0.11 (-0.01, 0.23)	0.082	0.0	0.495
≤4000	6	0.06 (-0.04, 0.17)	0.239	29.8	0.212
**Duration (wk)**					
≤14	4	0.06 (-0.07, 0.20)	0.360	0.0	0.701
>14	5	0.08 (-0.03, 0.20)	0.165	45.3	0.120
**Presence of cosupplementation**					
No	7	0.10 (-0.03, 0.23)	0.132	25.6	0.233
Yes	2	0.07 (-0.00, 0.15)	0.056	0.0	0.409
**Quality**					
Critically low	3	0.11 (-0.01, 0.23)	0.076	0.0	0.734
Low	5	0.06 (-0.12, 0.24)	0.519	44.6	0.124
High	1	0.09 (0.01, 0.17)	0.038	—	—
**Effect of vitamin D supplementation on HDL levels (WMD)**					
Overall	16	0.16 (-0.25, 0.57)	0.453	69.3	≤ 0.001
**Renal and liver disease**					
Yes	4	0.73 (-0.17, 1.64)	0.185	62.52	0.025
No	12	0.09 (-0.65, 0.84)	0.351	82.13	≤ 0.001
**Number of included studies**					
<10	12	0.28 (-0.24, 0.79)	0.288	58.5	0.005
≥10	4	0.03 (-0.90, 0.95)	0.951	78.5	0.003
**Sex**					
F	4	-0.45 (-0.74, -0.17)	0.002	0.0	0.989
M/F	12	0.60 (-0.04, 1.24)	0.068	73.0	≤ 0.001
**Age(y)**					
≤40	6	-0.02 (-0.19, 0.14)	0.799	0.0	0.524
>40	7	-0.08 (-0.82, 0.66)	0.831	70.6	0.002
NR	3	1.88 (0.94, 2.81)	≤ 0.001	0.0	0.763
**Dose (IU/d)**					
>4000	6	-0.30 (-0.84, 0.24)	0.276	0.0	0.630
≤4000	8	0.20 (-0.32, 0.71)	0.457	80.0	≤ 0.001
NR	2	2.32 (0.81, 3.83)	0.003	0.0	1.000
**Duration (wk)**					
≤14	7	0.33 (-0.60, 1.25)	0.486	62.2	0.014
>14	7	-0.11 (-0.55, 0.34)	0.634	73.3	≤ 0.001
NR	2	2.32 (0.81, 3.83)	0.003	0.0	1.000
**Presence of cosupplementation**					
No	11	-0.12 (-0.45, 0.21)	0.490	44.5	0.055
Yes	3	1.08 (-1.36, 3.51)	0.386	90.3	≤ 0.001
NR	2	2.32 (0.81, 3.83)	0.003	0.0	1.000
**Quality**					
Critically low	5	-0.70 (-1.23, -0.18)	0.009	0.0	0.703
Low	9	0.37 (-0.16, 0.89)	0.170	79.7	≤ 0.001
Moderate	1	-0.14 (-0.99, 0.71)	0.747	—	—
High	1	1.14 (-0.64, 2.92)	0.209	—	—

Abbreviations: CI, confidence interval; ES, effect size; HDL, high-density lipoprotein; LDL, low-density lipoprotein; MD, mean difference; NAFLD, nonalcoholic fatty liver disease; NR, not reported; PCOS, polycystic ovary syndrome; SMD, standardized mean difference; TC, total cholesterol; TG, triglyceride; WMD, weighted mean difference.

1 Obtained from random-effects model

2 Refers to the mean (95% CI)

3 Inconsistency, percentage of variation across studies due to heterogeneity

4 Obtained from Q-test

Sensitivity analysis revealed that the overall ES was independent of a single study. Neither Begg’s nor Egger’s tests revealed a statistically significant small-study effect (*P* = 0.386 and *P* = 0.304, respectively). However, publication bias was observed by visually examining the funnel plot. Therefore, trim-and-fill analysis was performed with 2 imputed studies, but the results did not change (ES: −0.14; 95% CI: −0.21, −0.07; *P* < 0.05) (Figure 2B).

#### Effect of vitamin D supplementation on TG levels in studies reporting WMD

**FIGURE 3:**
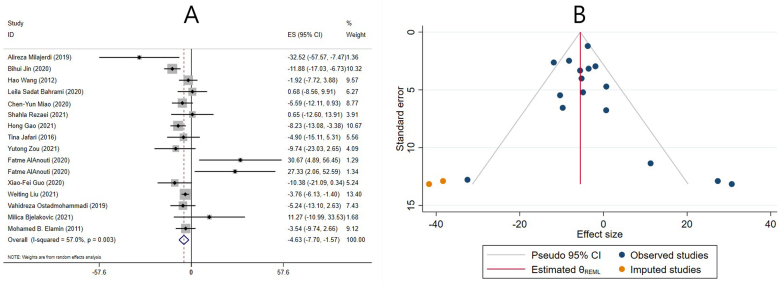
The effects of vitamin D supplementation on triglyceride levels. Forest plot (A) detailing weighted mean difference (WMD) and 95% confidence intervals (CIs); Trim-and-fill analysis (B). ES, effect size; REML, restricted maximum likelihood method.

The effect of vitamin D supplementation on TG levels was reported in 15 meta-analyses with 16 ESs, which reported their results based on the WMD. The combined ESs from these studies demonstrated a significant reduction in TG (ES: −4.63 mg/dL; 95% CI: −7.70, −1.57; *P* = 0.003) (Figure 3A). There was a significant between-study heterogeneity (I^2^ = 57.0%, *P* = 0.003) (Figure 3A). The number of included ESs, the age of the participants, dose, duration, and the use of cosupplements might be potential sources of heterogeneity. Subgroup analysis revealed that vitamin D supplementation significantly decreased TG levels in meta-analyses with ≥10 included ESs, females, participants ≤50 y old, individuals without renal and liver disease, >14 wk supplementation, and in meta-analyses that did not include any studies with cosupplements. Interestingly, only low-quality studies demonstrated a significant reduction in TG levels after vitamin D supplementation. Moreover, the 2 subcategories of vitamin D dose did not report a significant change in TG levels following the supplementation (Table 4). In addition, using subgroup analysis, the impact of renal and liver disease on overall ES was investigated. After excluding studies that evaluated liver and renal disease, the result remained unchanged, and vitamin D significantly reduced the TG levels (Table 4).

Sensitivity analysis revealed that the overall ES was independent of any study’s removal. Begg’s and Egger’s tests did not reveal a significant small-study effect (*P* = 0.224 and *P* = 0.474, respectively). A visual inspection of the funnel plot also revealed publication bias. Thus, trim-and-fill analysis was conducted with 2 imputed studies, with no change in the result (ES: −5.53 mg/dL; 95% CI: −8.14, −2.93; *P* < 0.05) (Figure 3B).

### Effect of vitamin D supplementation on TC levels

#### Effect of vitamin D supplementation on TC levels in studies reporting SMD

**FIGURE 4:**
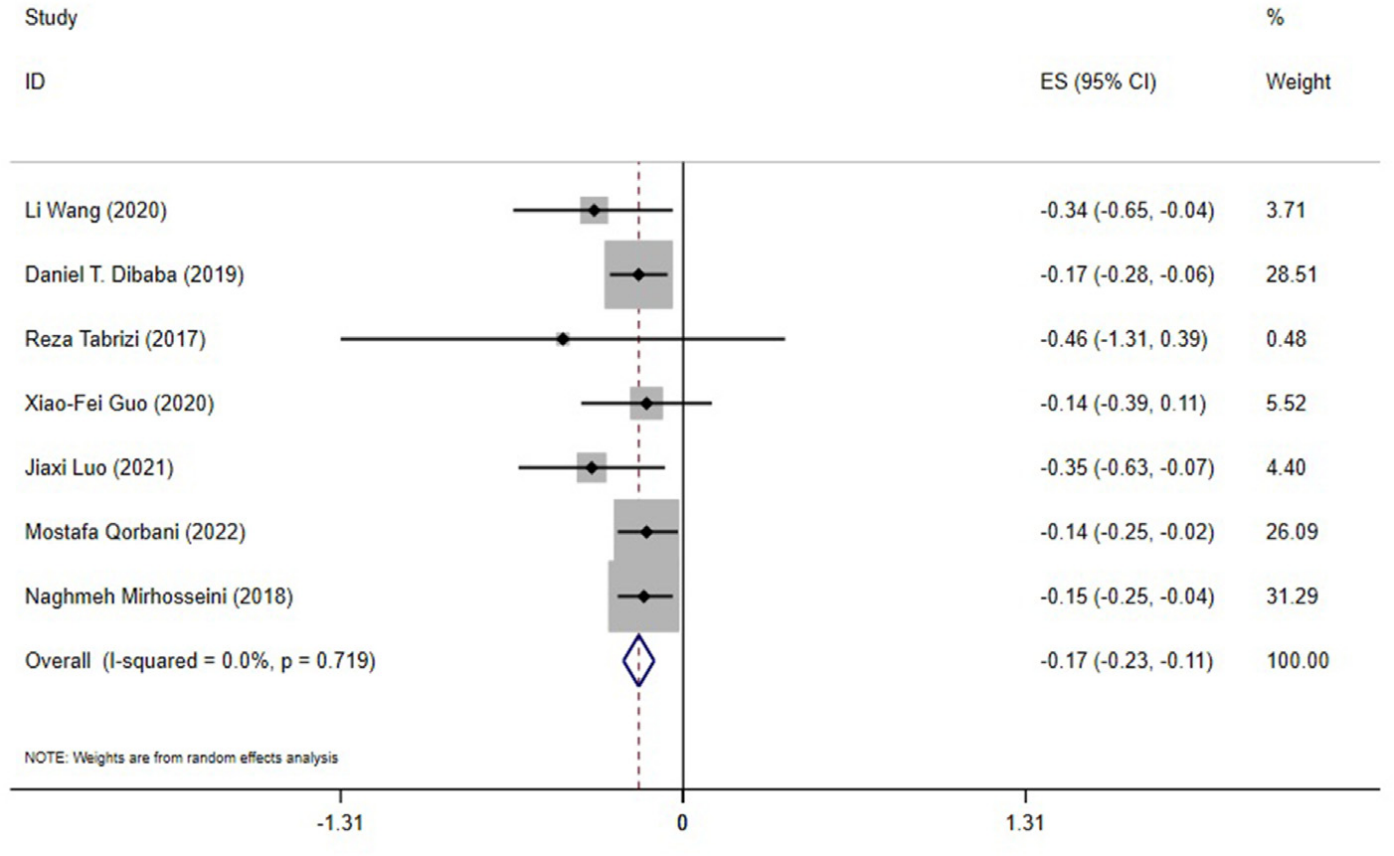
The effects of vitamin D supplementation on total cholesterol levels. Forest plot detailing standardized mean difference (SMD) and 95% confidence intervals (CIs). ES, effect size.

According to 7 meta-analyses based on the SMD, vitamin D supplementation significantly reduced TC levels (ES: −0.17; 95% CI: −0.23, −0.11; *P* ≤ 0.001) (Figure 4). There was no significant heterogeneity between studies (I^2^ = 0.0%, *P* = 0.719) (Figure 4). According to the subgroup analysis, vitamin D significantly decreased TC levels in individuals without renal and liver disease and in low- and high-quality studies. Vitamin D had a more robust effect on TC levels in meta-analyses that included <10 ESs, studies that only included females, studies with ≤50 y old participants, studies >4000 IU/d vitamin D supplementation, studies ≤14 wk of supplementation, and in meta-analyses that included one or more studies with cosupplements (Table 4). Moreover, subgroup analysis was performed to investigate the impact of renal and liver disease on overall ES. After excluding studies that evaluated liver and renal disease, the result remained unchanged, and vitamin D significantly reduced TC levels (Table 4).

Sensitivity analysis showed that no single study likely affected the overall ES. Due to the small number of included studies for this outcome, neither Begg’s nor Egger’s tests were conducted. In addition, the funnel plot was not conducted for the same reason.

#### Effect of vitamin D supplementation on TC levels in studies reporting WMD

**FIGURE 5:**
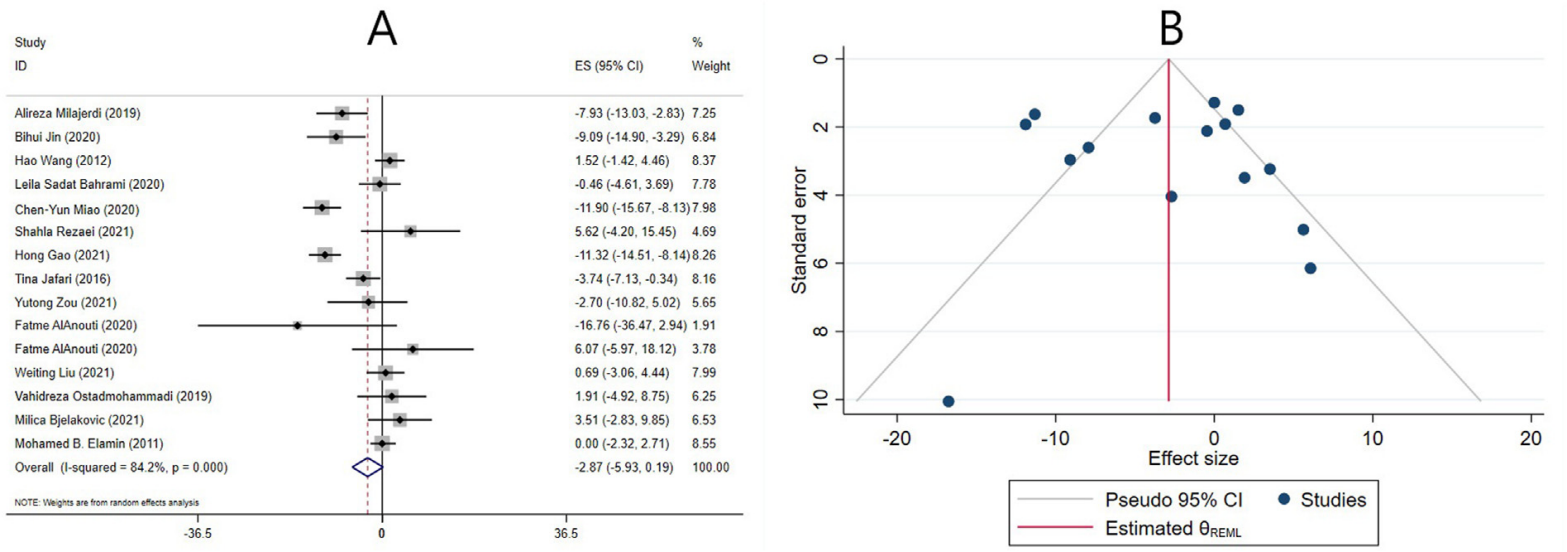
The effects of vitamin D supplementation on total cholesterol levels. Forest plot (A) detailing weighted mean difference (WMD) and 95% confidence intervals (CIs); Trim-and-fill analysis (B). ES, effect size; REML, restricted maximum likelihood method.

According to 14 meta-analyses with 15 ESs based on the WMD, vitamin D supplementation did not affect TC levels (ES: −2.87 mg/dL; 95% CI: −5.93, 0.19; *P* = 0.066) (Figure 5A). Significant heterogeneity existed between studies (I^2^ = 84.2%, *P* ≤ 0.001) (Figure 5A). Study quality, presence of cosupplementation, dose and duration of supplementation, and participant age could be considered as potential sources of heterogeneity. According to subgroup analysis, vitamin D significantly decreased TC levels in studies that included only females, those who were ≤50 y old, individuals without renal and liver disease, received >4000 IU/d or supplemented for ≤14 wk, and low-quality studies (Table 4). In addition, subgroup analysis was performed to investigate the impact of renal and liver disease on overall ES. After excluding studies that evaluated liver and renal disease, the result changed and reported that vitamin D significantly reduced TC levels (Table 4).

Sensitivity analysis showed that omitting Wang et al. [39], Rezaei et al. [37], AlAnouti et al. (higher dose) [20], Ostadmohammadi et al. [35], or Bjelakovic et al. [22] likely affected the overall ES, and TC levels significantly decreased after omitting one of the mentioned studies [20,22,35,37,39]. Begg’s and Egger’s tests revealed no small-study effect (*P* = 0.843 and *P*= 0.940, respectively). In addition, the asymmetric distribution of studies was not observed by visual inspection of the funnel plot (Figure 5B).

### Effect of vitamin D supplementation on LDL-C levels

#### Effect of vitamin D supplementation on LDL cholesterol levels in studies reporting SMD

**FIGURE 6:**
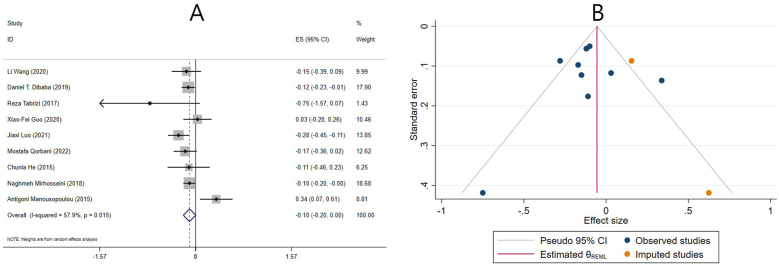
The effects of vitamin D supplementation on low-density lipoprotein cholesterol levels. Forest plot (A) detailing standardized mean difference (SMD) and 95% confidence intervals (CIs); Trim-and-fill analysis (B). ES, effect size; REML, restricted maximum likelihood method.

In the pooled results of 9 meta-analyses based on the SMD, supplementation with vitamin D did not significantly alter serum LDL cholesterol levels (ES: −0.10; 95% CI: −0.20, 0.00; *P* = 0.053) (Figure 6A). There was a substantial amount of heterogeneity (I^2^ = 57.9%, *P* = 0.015) (Figure 6A). Sex, age, study population, supplementation duration and dose, number of included ESs, and quality of studies were identified as sources of heterogeneity. Subgroup analysis revealed that vitamin D supplementation significantly decreased LDL cholesterol levels in meta-analyses that included ≥10 ESs, women with PCOS, in studies that only included females, in >50 y old individuals, when participants received ≤4000 IU/d, or were supplemented for ≤14 wk. In addition, in meta-analyses that included one or more studies with cosupplements and low-quality studies, LDL cholesterol levels were significantly reduced after vitamin D supplementation (Table 4). Moreover, subgroup analysis was performed to investigate the impact of renal and liver disease on overall ES. After excluding studies that evaluated liver and renal disease, the result remained unchanged, and vitamin D had no significant effect on LDL-C levels (Table 4).

Sensitivity analysis revealed that excluding the studies of Guo et al. [26] or Manousopoulou et al. [11] modified the overall effect, and LDL-C levels decreased significantly after excluding one of the studies mentioned above. Begg’s and Egger’s tests did not reveal any small-study effects (*P* = 1.000 and *P* = 0.972, respectively). Moreover, an examination of the funnel plot revealed an asymmetric distribution. Consequently, trim-and-fill analysis was carried out on 11 studies (2 imputed studies). Even after trim-and-fill analysis, the corrected ES for publication bias exhibited no significant effect (ES: −0.05; 95% CI: −0.17, 0.06; *P* > 0.05) (Figure 6B).

#### Effect of vitamin D supplementation on LDL cholesterol levels in studies reporting WMD

**FIGURE 7:**
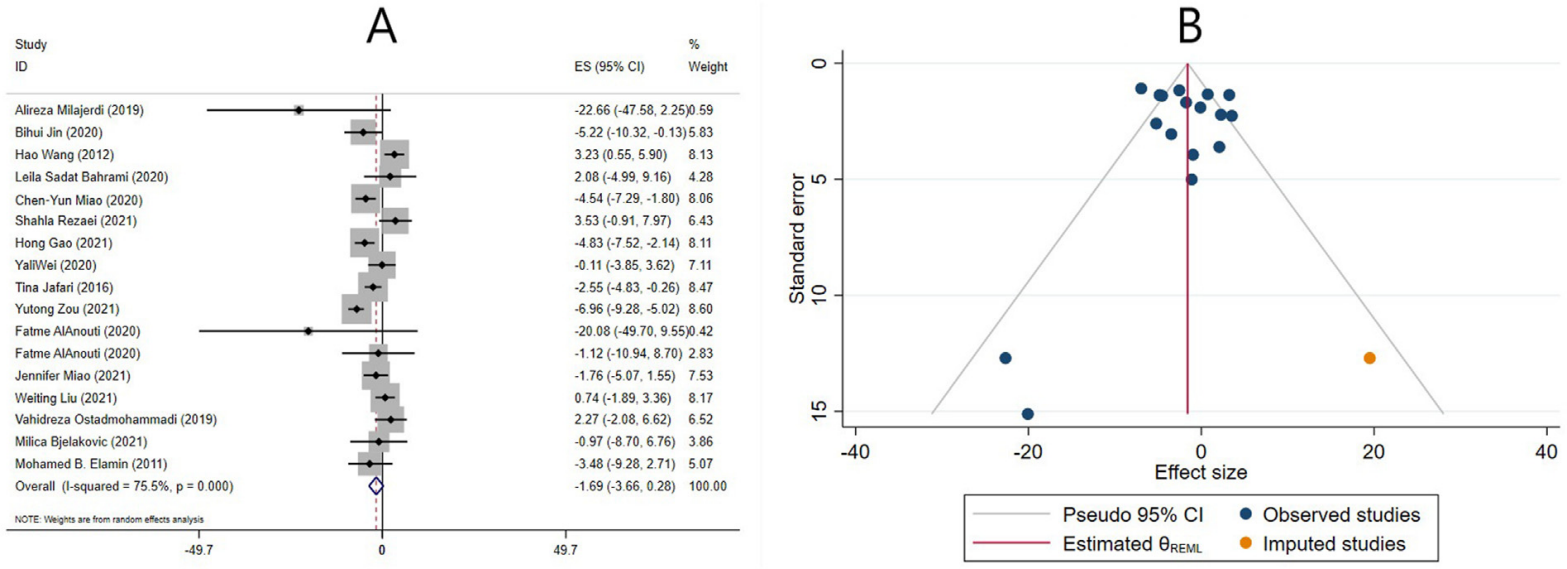
The effects of vitamin D supplementation on low-density lipoprotein cholesterol levels. Forest plot (A) detailing weighted mean difference (WMD) and 95% confidence intervals (CIs); Trim-and-fill analysis (B). ES, effect size; REML, restricted maximum likelihood method.

Vitamin D supplementation did not alter serum LDL cholesterol levels significantly (ES = -1.69 mg/dL; 95% CI: -3.66, 0.28, p = 0.092) in the pooled results of 16 meta-analyses with 17 ESs, based on the WMD (Figure 7A). There was a substantial amount of heterogeneity (I^2^= 75.5%, p ≤ 0.001) (Fig.7A). Age, supplementation duration and dose of studies, using cosupplements, and the quality of studies were identified as major sources of heterogeneity. Subgroup analysis revealed that supplementation with vitamin D significantly reduced LDL cholesterol levels in studies that only included females, individuals without renal and liver disease, ≤50 y old participants, individuals who received >4000 IU/d, were supplemented for ≤14 wk, and critically low-quality studies (Table 4). In addition, subgroup analysis was performed to investigate the impact of renal and liver disease on overall ES. After excluding studies that evaluated liver and renal disease, the result changed and reported that vitamin D significantly reduced LDL-C levels (Table 4).

The sensitivity analysis revealed that omitting Wang et al. [39] or Rezaei et al. [37] altered the overall effect, and LDL-C levels decreased significantly after omitting one of the studies. Begg’s and Egger’s tests yielded no statistically significant small-study effects (*P* = 1.000 and *P* = 0.807, respectively). In addition, a visual examination of the funnel plot revealed asymmetric distribution. Consequently, trim-and-fill analysis was conducted on 18 studies (1 imputed study). After correcting for publication bias, the results still remained nonsignificant (ES: −1.58 mg/dL; 95% CI: −3.43, 0.27; *P* > 0.05) (Figure 7B).

### Effect of vitamin D supplementation on HDL cholesterol levels

#### Effect of vitamin D supplementation on HDL cholesterol levels in studies reporting SMD

**FIGURE 8:**
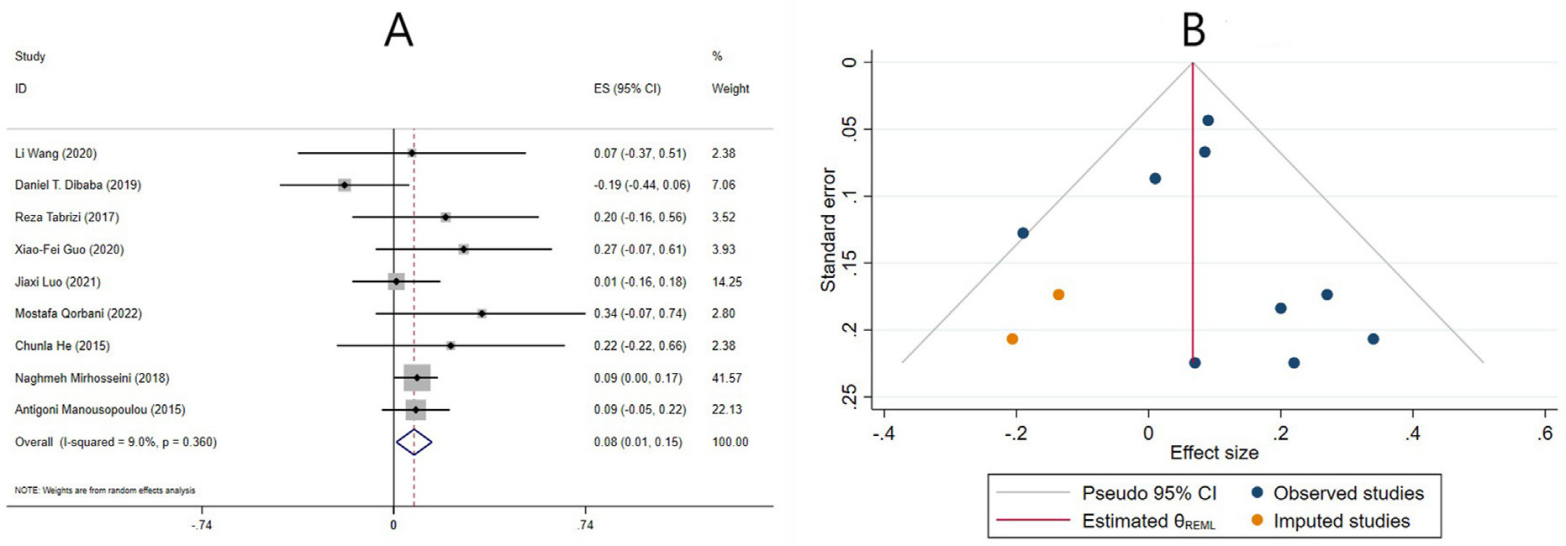
The effects of vitamin D supplementation on HDL cholesterol levels: Forest plot (A) detailing standardized mean difference (SMD) and 95% confidence intervals (CIs); Trim-and-fill analysis (B). ES, effect size; REML, restricted maximum likelihood method.

In 9 meta-analyses based on the SMD examining the effect of vitamin D supplementation on HDL cholesterol levels, the pooled ES revealed a significant increase in HDL cholesterol levels (ES: 0.08; 95% CI: 0.01, 0.15; *P* = 0.025) (Figure 8A). There was no evidence of significant heterogeneity between studies (I^2^ = 9.0%, *P* = 0.360) (Figure 8A). Subgroup analysis revealed that the after vitamin D supplementation, HDL cholesterol concentration was significantly increased in meta-analyses with <10 included studies, individuals without renal and liver disease, and those aged ≤40 y (Table 4). Moreover, subgroup analysis was performed to investigate the impact of renal and liver disease on overall ES. After excluding studies that evaluated liver and renal disease, the result remained unchanged, and vitamin D significantly increased HDL-C levels (Table 4).

After removing the studies of Tabrizi et al. [38], He et al. [27], Mirhosseini et al. [12], or Manousopoulou et al. [11] using sensitivity analysis, the ES was no longer statistically significant. Begg’s and Egger’s tests did not indicate the presence of a small-study effect (*P* = 0.675 and *P* = 0.582, respectively). In addition, publication bias was identified through a visual examination of the funnel plot. In light of this, a trim-and-fill analysis was conducted with 2 imputed studies, and the results remained statistically significant after adjusting for publication bias (ES: 0.06; 95% CI: 0.009, 0.125; *P* < 0.05) (Figure 8B).

#### Effect of vitamin D supplementation on HDL cholesterol levels in studies reporting WMD

**FIGURE 9:**
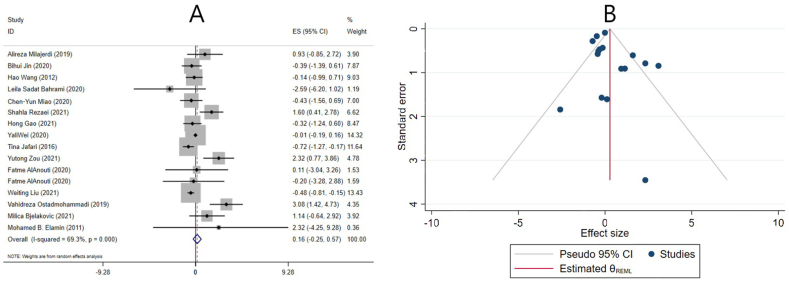
The effects of vitamin D supplementation on HDL cholesterol levels: Forest plot (A) detailing weighted mean difference (WMD) and 95% confidence intervals (CIs); Trim-and-fill analysis (B). ES, effect size; REML, restricted maximum likelihood method.

The effect of vitamin D supplementation on HDL cholesterol concentration was examined in 15 meta-analyses with 16 ESs based on the WMD. The pooled ES did not indicate a significant effect (ES: 0.16 mg/dL; 95% CI: −0.25, 0.57; *P* = 0.453) (Figure 9A). However, significant heterogeneity between studies was observed (I^2^ = 69.3%, *P* ≤ 0.001) (Figure 9A). Following subgroup analysis, the sex, age, dose and duration of vitamin D supplementation, presence of cosupplementation, and quality of the included ESs were identified as major sources of heterogeneity. Interestingly, subgroup analysis revealed that HDL cholesterol levels significantly decreased following vitamin D supplementation in studies that only included females and critically low-quality studies. Other subgroups did not report any significant changes in HDL cholesterol levels (Table 4). Moreover, subgroup analysis was performed to investigate the impact of renal and liver disease on overall ES. After excluding studies that evaluated liver and renal disease, the result remained unchanged and reported that vitamin D did not have any significant effect on HDL-C levels (Table 4).

After eliminating each study using sensitivity analysis, the ES remained nonsignificant. Begg’s and Egger’s tests did not indicate a small-study effect (*P* = 0.444 and *P* = 0.281, respectively). Moreover, publication bias was identified by visual examination of the funnel plot. Therefore, trim-and-fill analysis was performed with no imputed studies, and even after correcting for publication bias, the results remained nonsignificant (ES: 0.28 mg/dL; 95% CI: −0.30, 0.86; *P* > 0.05) (Figure 9B).

## Discussion

Contradictory findings have been reported regarding the effect of vitamin D on the lipid profile, an important factor in various diseases. Therefore, the present study was conducted to investigate this topic in greater depth and detail. The findings of this umbrella meta-analysis support the theory that vitamin D supplementation benefits the lipid profile and is advantageous as a dietary intervention in managing dyslipidemia. According to this study, vitamin D significantly decreased TG levels while having no significant effect on LDL cholesterol levels. In addition, the effects of vitamin D on HDL cholesterol and TC levels produced contradictory findings. Subgroup analyses suggested that doses ≥ 4000 IU/d and duration of intervention ≤12 wk may be key variables.

The current investigation indicated that vitamin D significantly decreased TG levels according to WMD and SMD analyses. In subgroup analysis, the WMD and SMD analyses yielded comparable results, such as a significant drop in TG levels in participants ≤50 y old following vitamin D supplementation. Nevertheless, there were some differences. In the SMD analysis, vitamin D supplementation significantly decreased TG levels in studies involving both sexes and both subcategories of vitamin D dose and duration, with >4000 IU/d supplementation dose and ≤14 wk intervention having a more robust effect. In the WMD analysis, vitamin D supplementation decreased TG levels significantly only in females and those receiving supplementation for >14 wk. Two studies by Bjelakovic et al. [22] and AlAnouti et al. [20], which reported their analysis based on WMD, largely contributed to these variations between SMD and WMD analysis. These 2 studies also had an important role in the nonsignificant results of some subgroup analyses, such as sex, age, dose, and duration of vitamin D supplementation. Bjelakovic et al. [22] discovered no effect but a tendency for TG levels to rise after supplementation with vitamin D. This study included at least one study that used cosupplements with vitamin D, which might be one of the reasons for the inconsistent results. This hypothesis was more supported when subgroup analysis revealed that vitamin D significantly decreased TG levels only in meta-analyses where cosupplements were not administered. AlAnouti et al. [20] reported that both low and high doses of vitamin D supplementation significantly increased TG levels. This study had a high concentration of heterogeneity, and only 2 studies were included in each subgroup of supplementation dose. In addition, the intervention group’s baseline TG levels were significantly higher than those of the control group, which may affect the precision of the reported results.

Regarding TC, the levels of this lipid profile component decreased significantly following vitamin D supplementation according to SMD but not WMD analysis. However, in the WMD analysis, after excluding the articles that were performed on individuals with liver and renal disease, results changed, and a significant reduction in TC levels was reported. This may be due to the role of these organs in activating vitamin D in the body, and a disorder in these organs may reduce the effectiveness of vitamin D supplementation in correcting vitamin D deficiency and restoring proper function. The studies in the WMD analysis exhibited a high degree of heterogeneity. One of the potential causes of this heterogeneity might be due to the study by AlAnouti et al. [20], which reported 2 contradictory nonsignificant results for TC levels following a higher and lower dose of vitamin D supplementation. As mentioned previously, this study combined a small number of ES studies in each subgroup, reducing the accuracy and generalizability of the overall outcome. Studies by Wang et al. [39], Rezaei et al. [37], Ostadmohammadi et al. [35], Bjelakovic et al. [22], and AlAnouti et al. [20] in its higher dose subgroup found no effect but a tendency for TC levels to rise in their research. Omitting any one of the mentioned studies indicated that vitamin D supplementation significantly decreased TC levels in WMD analysis. Except for the study by AlAnouti et al. [20], these studies had a lower vitamin D supplementation dose (≤ 4000 IU/d), which might be a significant factor in the lack of efficacy of vitamin D in lowering TC levels. Moreover, some of the RCTs included in the study by Rezaei et al. [37] were stated to have critical biases, which should be considered when reviewing this article.

The current umbrella review reported that vitamin D did not significantly affect LDL cholesterol levels in both SMD and WMD analyses. In the SMD analysis, an interesting result was reported. In a meta-analysis by Manousopoulou et al. [11], vitamin D was found to significantly increase LDL cholesterol, contradicting our hypothesis and other similar studies. In addition, the study by Guo et al. [26] revealed that vitamin D had no effect but a tendency to raise LDL cholesterol levels. After excluding one of these studies [11,26], the findings indicated that vitamin D significantly reduced LDL cholesterol levels in the SMD analysis. The reason for this increase in LDL cholesterol levels after vitamin D supplementation in studies by Guo et al. and Manousopoulou et al. needs to be clarified. Significant biases were observed in the RCTs included in these meta-analyses; the quality of these meta-analyses was low to critically low, and the number of RCTs included in these meta-analyses was few, which may have compromised the reliability of the results.

The WMD analysis also yielded comparable results. According to the meta-analysis conducted by Wang et al. [39], vitamin D significantly increased LDL cholesterol levels. In addition, Bahrami et al. [21], Rezaei et al. [37], Liu et al. [30], and Ostadmohammadi et al. [35] reported that vitamin D supplementation had no effect but a tendency to increase LDL cholesterol levels. These meta-analyses had 2 similarities: a lower dose of vitamin D supplementation (≤4000 IU/d) and a supplementation duration of >14 wk. An extended duration of supplementation may introduce biases, and one of the major contributors is a decreased adherence rate to the intervention. In this regard, in the WMD section, the results reported that vitamin D significantly reduced LDL cholesterol levels in studies with >4000 IU/d and studies with ≤14 wk of supplementation. In addition, after excluding the articles that were performed on individuals with liver and renal disease, results changed, and a significant reduction in LDL cholesterol levels was reported. As previously mentioned, this may be due to these organs’ roles in activating vitamin D.

The results varied regarding the effect of vitamin D supplementation on HDL cholesterol levels. Based on SMD analysis, vitamin D supplementation resulted in a significant increase in HDL cholesterol levels, in contrast to the WMD analysis. Notably, 2 studies conducted by Jafari et al. [28] and Liu et al. [30] found a significant decrease in HDL cholesterol levels following vitamin D supplementation. In these 2 studies, vitamin D supplementation was administered at lower doses (≤4000 IU/d) and for longer durations (>14 wk). As previously mentioned, the prolonged duration of supplements may introduce bias. In addition, according to the meta-analysis conducted by Jafari et al., in some of the included RCTs, despite supplementation with vitamin D, the participants’ deficiencies had not been corrected at the end of the trial. Bahrami et al. [21] conducted a meta-analysis on patients with coronary artery disease and low levels of 25(OH)D. Vitamin D supplementation was found to increase 25(OH)D levels in 2 included RCTs. Bahrami et al., however, reported no effect but a tendency for HDL cholesterol levels to decrease following vitamin D supplementation. Due to the small sample size, the reported results may not be reliable. Therefore, these flaws, along with the low and critically low-quality of the mentioned studies, may account for the nonsignificant results in the WMD analysis. Furthermore, in the studies that reported WMD, the interesting point was that vitamin D did significantly decrease HDL cholesterol levels in the RCTs in which only females participated. There were 4 studies in this subgroup, one of which from Liu et al. [30]. The other 3 studies were by Gao et al. [25], Jin et al. [29], and Miao et al. [32]. These 3 meta-analyses focused solely on RCTs that involved people with PCOS. Vitamin D supplementation might exert its biological impacts differently in various health conditions, like PCOS, which high-quality studies should investigate to determine whether this hypothesis is true. In addition, as reported in Table 2, only 5 of the 10 included RCTs in the meta-analysis by Gao et al. [25] were evaluated to have high quality. There were concerns about selection, detection, and attrition biases in the included RCTs. Therefore, these flaws may have led to biased results.

The following mechanisms may explain how vitamin D affects lipid profile levels: *1*) By promoting calcium absorption, vitamin D may inhibit the absorption of fatty acids. Comparable interactions between calcium and bile acids strengthen this effect. The interaction between calcium and bile acids may reduce the quantity of bile acid available to facilitate fat absorption in the intestinal tract [60]. *2*) Vitamin D might improve the lipid profile by reducing insulin resistance [61]. *3*) By increasing peroxisome proliferator-activated receptor (PPAR)-γ expression, which increases insulin synthesis and release, and possibly by promoting insulin receptor expression or suppressing proinflammatory cytokines, vitamin D may reduce insulin resistance, thereby affecting the lipid profile [[62], [63], [64]]. *4*) By increasing PPAR-α expression, vitamin D may significantly impact lipid profile metabolism [65]. *5*) Vitamin D may increase lipoprotein lipase activity and gene expression in muscles and adipose tissue, thereby enhancing the clearance of lipoprotein particles from circulation and altering the lipid profile to reduce atherosclerosis. The most noticeable effect of lipoprotein lipase (LPL) is a reduction in serum TGs and an increase in serum HDL [66]. *6*) Elevated parathyroid hormone (PTH) concentrations can reduce plasma post-heparin lipolytic activity; thus, the suppressive effect of vitamin D on serum PTH concentrations may reduce serum TGs via increased peripheral removal [67]. *7*) Vitamin D may regulate macrophage function on reverse cholesterol transport and large HDL particles; in this case, serum TG levels might decrease [68]. *8*) Insulin-induced gene-2 (Insig-2) inhibits cholesterol synthesis by downregulating sterol regulatory-element binding protein-2 activation and 3-hydroxy-3-methylglutaryl-coenzyme A reductase expression. Vitamin D can affect this pathway via its effect on the transcriptional activity of VDR and Insig-2 expression [69]. *9*) According to an experimental study, vitamin D may reduce TG deposition in differentiated adipocytes, increase fatty acid β-oxidation, and decrease de novo fatty acid synthesis [70]. *10*) Vitamin D may affect the lipid profile by modifying Apo B100 (the major component of LDL cholesterol) and Apo A1 levels (the major component of HDL cholesterol). However, studies have reported inconsistent results [71,72]. A meta-analysis found that vitamin D had no significant effect on these apolipoproteins [73].

The present study is the first umbrella meta-analysis investigating the impact of vitamin D supplementation on lipid profile. In all variables, no small-study effect was observed. Furthermore, in case of publication bias, the significant or nonsignificant results of none of the lipid profiles changed after correction by trim-and-fill. The quality of studies was checked using the AMSTAR2 tool**.** Basically, meta-analyses cannot pool RCTs with cosupplements with the ones without. However, in some meta-analyses, this criterion was not considered in their study selection, and at least one RCT with cosupplement was included. Therefore, our subgroup analyses attempted to adjust this issue to some extent. However, there were some limitations. Included studies should have considered the initial 25(OH)D and lipid profile levels, whether there are within the normal range or not, which most did not. Furthermore, some studies did not consider topics such as altitude, race, body fat, and BMI, which can affect the relationship between vitamin D and lipid profile. Therefore, we could not evaluate these variables and determine their effects. The meta-analyses included RCTs comparing calcitriol, ergocalciferol, cholecalciferol, and alfacalcidol to placebo or no treatment, with some studies not even reporting the type of vitamin D supplement. Therefore, despite our best efforts and as per the protocol, we could not separate the effects of the various vitamin D supplements.

## Conclusion

This umbrella meta-analysis reported that vitamin D might decrease TG levels. It may also decrease TC levels in individuals who were supplemented with a dose of >4000 IU/d or for ≤14 wk. Moreover, after statistical analyses, it can be suggested that vitamin D might decrease LDL cholesterol levels. Regarding HDL cholesterol, we could not make a clear conclusion. However, due to the high heterogeneity of some of our results and the poor quality of the included meta-analyses, results should be interpreted with caution. In addition, although the results are statistically significant, the clinical significance may not be considerable. The present study supports that vitamin D supplementation could be considered a beneficial adjuvant therapy in managing lipid profile levels, especially in individuals with vitamin D deficiency.

## Author contributions

The authors’ responsibilities were as follows—NR: conceived the study, developed the criteria, conducted the systematic search, screened articles, extracted data, and drafted and revised the manuscript; MZ: performed analysis, conducted the systematic search, screened articles, extracted data, assisted in study design, and revised the manuscript; PJ: assisted in the study design and revised the manuscript; AO conceived the study, developed the criteria, provided content expertise, and revised the manuscript; and all authors: read and approved the final version.

## Conflicts of interest

The authors report no conflicts of interest.

## Funding

The authors reported no funding received for this study.

## Data availability

Data described in the manuscript, code book, and analytic code will be made available upon request pending.

## Appendix A Supplementary data

Multimedia component1 Multimedia component1
