# Supplementary material for: The Effect of Vitamin D Supplementation on Lipid Profiles: an Umbrella Review of Meta-Analyses
Source: Adv Nutr. 2023 Aug 30;14(6):1479–98. doi: 10.1016/j.advnut.2023.08.012 (PMC10721514; doi:10.1016/j.advnut.2023.08.012)
Supplement: Multimedia component1 [file mmc1.docx]

**PubMed**

TI/AB:

"vitamin d"[Title/Abstract] OR "ergocalciferols"[Title/Abstract] OR ("vitamin"[Title/Abstract] AND "d2"[Title/Abstract]) OR "vitamin d2"[Title/Abstract] OR "cholecalciferol"[Title/Abstract] OR ("vitamin"[Title/Abstract] AND "d3"[Title/Abstract]) OR "vitamin d3"[Title/Abstract] OR "cholecalciferols"[Title/Abstract] OR "colecalciferol"[Title/Abstract] OR "ergocalciferol"[Title/Abstract] OR "alfacalcidol"[Title/Abstract] OR "alphacalcidol"[Title/Abstract] OR "calcitriol"[Title/Abstract] OR "calcitriols"[Title/Abstract] OR "25(OH)D"[Title/Abstract] OR "25(OH)D2"[Title/Abstract] OR "25(OH)D3"[Title/Abstract] OR "calcifediol"[Title/Abstract] OR "25 hydroxycholecalciferol"[Title/Abstract] OR "25-hydroxyvitamin D"[Title/Abstract] OR "25 hydroxyvitamin d"[Title/Abstract] OR "25 hydroxyvitamin d3"[Title/Abstract] OR "25-hydroxyvitamin d 2"[Title/Abstract] OR "25 hydroxyvitamin d2"[Title/Abstract] OR "dihydrotachysterol"[Title/Abstract] OR "dihydroxycholecalciferols"[Title/Abstract] OR "dihydroxycholecalciferol"[Title/Abstract] OR "1,25-dihydroxyvitamin D"[Title/Abstract] OR "1,25 dihydroxyvitamin d"[Title/Abstract] OR ("1,25"[Title/Abstract] AND "dihydroxyvitamin"[Title/Abstract] AND "d3"[Title/Abstract]) OR "1,25 dihydroxyvitamin d3"[Title/Abstract] OR "1,25-dihydroxyergocalciferol"[Title/Abstract] OR "1,25 dihydroxyvitamin d2"[Title/Abstract] OR "calciferol"[Title/Abstract] OR "calciferols"[Title/Abstract] OR "paricalcitol"[Title/Abstract] OR "1 alpha-hydroxyergocalciferol"[Title/Abstract] OR "doxercalciferol"[Title/Abstract] OR "calcidol"[Title/Abstract] OR "calcifediol"[Title/Abstract] OR "calcidiol"[Title/Abstract] OR "hydroxycholecalciferols"[Title/Abstract] OR "hydroxycholecalciferol"[Title/Abstract] OR "hydroxyergocalciferol"[Title/Abstract] OR "rocaltrol"[Title/Abstract] OR "drisdol"[Title/Abstract] OR "vectical"[Title/Abstract]

Mesh:

"vitamin d"[MeSH Terms] OR "ergocalciferols"[MeSH Terms] OR "cholecalciferol"[MeSH Terms] OR "alfacalcidol"[Supplementary Concept] OR "calcitriol"[MeSH Terms] OR "calcifediol"[MeSH Terms] OR "25-hydroxyvitamin D"[Supplementary Concept] OR "25-hydroxyvitamin d 2"[MeSH Terms] OR "dihydrotachysterol"[MeSH Terms] OR "dihydroxycholecalciferols"[MeSH Terms] OR "1,25-dihydroxyvitamin D"[Supplementary Concept] OR "1,25-dihydroxyergocalciferol"[Supplementary Concept] OR "paricalcitol"[Supplementary Concept] OR "1 alpha-hydroxyergocalciferol"[Supplementary Concept] OR "hydroxycholecalciferols"[MeSH Terms]

TI/AB:

"LDL"[Title/Abstract] OR "HDL"[Title/Abstract] OR "TC"[Title/Abstract] OR "TG"[Title/Abstract] OR "TAG"[Title/Abstract] OR "total cholesterol"[Title/Abstract] OR "cholesterol"[Title/Abstract] OR "cholesterol's"[Title/Abstract] OR "cholesterole"[Title/Abstract] OR "cholesterols"[Title/Abstract] OR "triglycerid"[Title/Abstract] OR "triglycerides"[Title/Abstract] OR "triglyceride"[Title/Abstract] OR "triglycerids"[Title/Abstract] OR "triacylglycerol"[Title/Abstract] OR "triacylglycerols"[Title/Abstract] OR ("lipoproteins"[Title/Abstract] AND "ldl"[Title/Abstract]) OR "ldl lipoproteins"[Title/Abstract] OR ("low"[Title/Abstract] AND "density"[Title/Abstract] AND "lipoprotein"[Title/Abstract]) OR "low density lipoprotein"[Title/Abstract] OR ("lipoproteins"[Title/Abstract] AND "hdl"[Title/Abstract]) OR "hdl lipoproteins"[Title/Abstract] OR ("high"[Title/Abstract] AND "density"[Title/Abstract] AND "lipoprotein"[Title/Abstract]) OR "high density lipoprotein"[Title/Abstract] OR "lipoprotein's"[Title/Abstract] OR "lipoproteine"[Title/Abstract] OR "lipoproteins"[Title/Abstract] OR "lipoprotein"[Title/Abstract] OR "lipid profile"[Title/Abstract] OR "LDL-c"[Title/Abstract] OR "LDL-cholesterol"[Title/Abstract] OR "HDL-c"[Title/Abstract] OR "HDL-cholesterol"[Title/Abstract]

Mesh:

Cholesterol[MeSH Terms] OR triglycerides[MeSH Terms] OR "lipoproteins, ldl"[MeSH Terms] OR "lipoproteins, hdl"[MeSH Terms] OR "lipoproteins"[MeSH Terms] OR "Cholesterol, LDL"[Mesh] OR "Cholesterol, HDL"[Mesh]

------------------------------------------------------------------------------------------------------------------------------

TI/AB:

"meta-analysis"[Title/Abstract] OR "meta-analyses"[Title/Abstract]

Mesh:

"meta-analysis"[Publication Type] OR "meta-analysis as topic"[MeSH Terms]
